# Nitrate containing vegetables and dietary nitrate and nonalcoholic fatty liver disease: a case control study

**DOI:** 10.1186/s12937-023-00834-z

**Published:** 2023-01-10

**Authors:** Parvin Mirmiran, Farshad Teymoori, Hossein Farhadnejad, Ebrahim Mokhtari, Ammar Salehi-Sahlabadi

**Affiliations:** 1 Nutrition and Endocrine Research Center, Research Institute for Endocrine Sciences, Shahid Beheshti University of Medical Sciences, Tehran, Iran; 2 Department of Nutrition, School of Public Health, Iran University of Medical Sciences, Tehran, Iran; 3 Department of Clinical Nutrition and Dietetics, Facualty of Nutrition and Food Technology, National Nutrition and Food Technology, Shahid Beheshti University of Medical Sciences, Tehran, Iran

**Keywords:** Nitrate, Nitrate-containing vegetables, Nonalcoholic fatty liver diseases, NAFLD

## Abstract

**Background:**

Vegetables is the main sources of dietary nitrate. Studies suggested the potential link between nitrate content of vegetables and reduce the risk of chronic diseases. We aimed to assess the association between nitrate-containing vegetables (NCVs) with odds of nonalcoholic fatty liver diseases (NAFLD) in Iranian adults.

**Method:**

This case-control study was performed on a total of 225 newly diagnosed NAFLD cases and 450 controls aged 20–60 years. Individuals’ dietary intakes were determined using a valid and reliable food frequency questionnaire.

**Results:**

The mean ± SD age and BMI of participants were 38.1 ± 8.8 years and 26.8 ± 4.3 kg/m^2^, respectively. In the fully adjusted model, the odds of NAFLD were decreased across tertiles of total NCVs [(adjusted OR: 0.20, 95%CI: 0.10–0.40), (P_trend_ <  0.001)] and low-nitrate vegetables [(adjusted OR: 0.22, 95%CI: 0.11–0.48), (P_trend_ <  0.001)]. Our results showed that each one SD increments in nitrate content of vegetables (adjusted OR: 0.73, 95%CI: 0.55–0.97) and nitrate content of fruits (adjusted OR: 0.59, 95%CI: 0.36–0.97) was associated with reduced odds of NAFLD (*P* <  0.05). However, there was a positive association between each one SD increments in nitrate content of dairy products and meats and processed meats with odds of NAFLD (adjusted OR: 1.34, 95%CI: 1.03–1.74), (*P* <  0.05).

**Conclusion:**

Our finding suggested that a higher intake of vegetable nitrate may be related to a decrease the odds of NAFLD.

## Background

Nonalcoholic fatty liver disease (NAFLD) is one of the major global health challenges that include many liver abnormalities such as hepatic steatosis, cirrhosis, or hepatocellular carcinoma [1]. This chronic disease is characterized by the accumulation of more than 5% of fat in the liver tissue cells in the absence of other risk factors; virus, immune or metabolic disorders, and drug abuse [2]. The Worldwide estimated prevalence of NAFLD in the general population is 25% [3]. Also, the prevalence of NAFLD among Iranian children and adults has been 7 and 35%, respectively [4]. Along with genetic predisposition, modifiable lifestyle factors, including physical inactivity, high body mass index (BMI), smoking, and unhealthy dietary pattern as prominent risk factors, can play an important role in the progression, development, and prevention of NAFLD [5–7].

Based on previous evidence, higher adherence to a healthy dietary pattern, characterized by higher intakes of vegetable, fruit, and whole-grain and lower intakes of red and processed meat, is one of the helpful nutritional strategies to prevent or delay the onset of NAFLD [8–10]. In addition to the nutrients contents of the food mentioned above groups, some other compounds of these foods, such as nitrate and nitrite, have been considered by health researchers regarding their impact on health and the risk of disease [8, 11–13]. Vegetables and drinking water are the main sources of exogenous nitrate. Also, processed meat and animal food products are the main sources of exogenous nitrite [14]. The previous report has suggested that nearly 80–95% of dietary nitrate is derived from vegetable intakes, especially green leafy vegetables including spinach, lettuce, rocket, cabbage, radish, and red beetroot [14].

The evidence has reported that nitrate and nitrite, as the main source of nitric oxide (NO), can play various important biological roles through the nitrate-nitrite-NO pathway in the progression or development of chronic diseases [15]. In this regard, the findings of recently conducted studies on the role of dietary nitrate, especially nitrate-containing vegetables, in health and disease suggested the potential link between nitrate content of vegetables and reduce the risk of diabetes [11], chronic kidney disease (CKD) [16], atherosclerosis and ischemic cerebrovascular disease [17], blood pressure [8], and cardiovascular disease (CVD) [18], which share common metabolic parameters with NAFLD. However, to the best of our knowledge, the association between consumption of nitrate-containing vegetables and risk of development NAFLD have not yet been investigated, and only a recent experimental study has shown that dietary nitrate from vegetables can prevent hepatic steatosis. In contrast, nitrate from protein sources such as meats may increase the risk of liver disease [19].

Considering the lack of convincing evidence regarding the association of total dietary nitrate intake or nitrate-rich foods with liver function, in this study, we aimed to investigate the association of all dietary nitrate intake, nitrate-containing vegetables (NCVs), and also nitrate intake from other foods with the odds of NAFLD in the Iranian population.

## Materials and methods

### Study population

The work was performed in the Metabolic Liver Disease Research Center as a referral center affiliated to Isfahan University of Medical Sciences. Totally 225 newly diagnosed NAFLD patients and 450 controls aged 20–60 years were included for final analysis. The study details were previously reported [20]. Participants for NAFLD group was ascertained using the liver ultrasonography scan (USG) and confirmation by gastroenterologists. The control group was selected based on liver ultrasound from individuals who had no stage of liver steatosis. Our inclusion criteria were having no history of renal and hepatic diseases (Wilson’s disease, autoimmune liver disease, hemochromatosis, virus infection, and alcoholic fatty liver (, CVD, diabetes, malignancy, thyroid disorder, and autoimmune, not following a specific diet (due to a particular disease or weight loss) and not using potentially hepatotoxic or steatogenic drugs. Participants who completed less than 35 items of the food frequency questionnaire (FFQ) and those who under or over-reported daily energy intake (≤800 or ≥ 4500 kcal/d) were excluded.

### Dietary assessment

A previously validated and reliable semi-quantitative 168 items food frequency questionnaire (FFQ) was used to assessing the dietary intakes [21]. Skilled dietitians while they were unaware about NAFLD status, asked participants to report their average dietary intake during the last year as follow: never or less than once a month, 3–4 times per month, once a week, 2–4 times per week, 5–6 times per week, once daily, 2–3 times per day, 4–5 times per day, and 6 or more times a day. Standard Iranian household measures were used for converting each food item portion size into a gram scale [22]. Daily energy and nutrients intake were computed using the United States Department of Agriculture’s (USDA) Food Composition Table (FCT). Moreover, Iranian FCT was used for some local foods not listed in USDA FCT [23].

### Physical activity and anthropometric measurements

Physical activity levels were recorded through face-to-face interviews using the International Physical Activity Questionnaire (IPAQ). All measurements were expressed as Metabolic Equivalents per week (METs/week).

Participants’ weight and height was recorded by standard protocols. BMI was calculated as weight (kg) divided by height in square meters (*m*^2^).

### Assessment of other variables

Information about age, sex, marital status, socioeconomic status (SES), and smoking status were gathered via a demographic questionnaire. Three variables, including family size (≤4, > 4 people), education (academic and non-academic education), and acquisition (house ownership or not), were used to compute the SES score. An SES score of 3 equated to high, 2 was scored as moderate, and 1 or 0 was considered low.

### Determination of NCVs and nitrate intake

In the present study, NCVs were determined according to the Bahadoran et al. study [24]. Briefly, they calculated the nitrate contents of 87 food items, including grains, legumes, fruits and vegetables, dairy products, meats, and processed meats using validated spectrophotometric methods. NCVs were categorized as low-nitrate vegetables (< 50 mg/100 g fresh weight of vegetables, including potato, broad beans, tomato, ketchup, cucumber, squash, eggplant, string beans, carrot, garlic, onion, pepper, mushroom, and watermelon), medium-nitrate vegetables (50–100 mg/100 g fresh weight of vegetables, including cabbage and turnip), and high-nitrate vegetables (> 100 mg/100 g fresh weight of vegetables, including celery, lettuce, and spinach) [8]. Total nitrate intake was calculated from all nitrate-containing food items using the previous report by Bahadoran et al. study [24]. Also, nitrate intake from 4 food categories including vegetables, fruits, the sum of grains, legumes, and nuts, and the sum of dairy products and meats and processed meats were calculated.

### Statistical analysis

Statistical analysis was conducted using Statistical Package Software for Social Science, version 21 (SPSS Inc., Chicago, IL, USA). The normality of variables was tested by the Kolmogorov-Smirnov’s and histogram char. Baseline characteristics and dietary intakes were expressed as mean ± SD or median (25–75 interquartile range) for quantitative variables and number and percentages for qualitative variables. Independent sample t-test and chi-square were used to examine the differences between cases and controls for continuous and categorical variables, respectively. Participants were categorized into tertiles based on the total intake of NCVs. The general and dietary data across tertiles of NCVs intake were reported, and P for the trend of the continuous and categorical variables were computed using linear regression (median values of NCVs and total dietary nitrate intakes in each tertile as the independent variable and continuous variables as dependent variable) and chi-square test across tertiles of NCVs intake. Multivariable logistic regression was performed to assess the relationship between NCVs intake and its subgroups including low, medium, and high nitrate-containing vegetables and the NAFLD odds. Final models were adjusted for potential confounding variables, including age, sex, BMI, physical activity, smoking, SES, energy intake, dietary intake of saturated fatty acids, vitamin E, fiber, and fructose. The odds ratio (OR) and 95% confidence interval (CI) of NAFLD across tertiles of NCVs intake and its subgroups including low, medium, and high nitrate-containing vegetables and were reported. we also conducted additional multivariate logistic regression analyses to assess the association between increasing each standard deviation (SD) of total nitrate intake and nitrate consumed from dietary food groups including vegetables, fruits, the sum of grains, legumes, and nuts, and the sum of dairy products and meats and processed meats with the odds of NAFLD. *P*-values < 0.05 were considered statistically significant.

## Results

The mean ± SD age of the participants (53% Men) were 38.1 ± 8.8 years. The mean intake of NCVs and total dietary nitrate were 108.0 ± 69.3 g/d and 464.0 ± 175.0 mg/d, respectively. According to Table 1. the mean age and dietary intakes of protein, fiber, fructose, vitamin E, and fruits were significantly increased across tertiles of NCVs (P_trend_ <  0.05). However, The percentage of men were significantly decreased across tertiles of NCVs (P_trend_ <  0.05). Also, intakes of energy, whole grains, refined grains, fruits, high-fat dairy products, and red and process meat were decreased across tertiles of NCVs (P_trend_ <  0.05).

**Table 1:** Characteristics and dietary intakes across tertiles of total intake of nitrate-containing vegetables among the study population

	Tertiles of NCVs			P-trend
	T1(***n*** = 230)	T2(***n*** = 225)	T3(***n*** = 220)	
Age(year)	37.0 ± 8.3	38.2 ± 8.5	39.5 ± 9.6	0.002
Male, n (%)	160 (61.5)	114 (51.1)	84 (43.8)	0.001
BMI(Kg/m^2^)	26.8 ± 4.1	26.7 ± 4.4	26.9 ± 4.4	0.687
Smoking, n (%)	9 (3.5)	11 (4.9)	8 (4.2)	0.722
Physical activity (MET/min/week)	1462 ± 862	1454 ± 850	1368 ± 939	0.261
SES, n(%)				0.638
Low	100 (38.5)	88 (39.5)	70 (36.5)	
Middle	90 (34.6)	87 (39.0)	75 (39.1)	
High	70 (26.9)	48 (21.5)	47 (24.5)	
**Dietary intake**				
**Macro and micronutrients**				
Energy intake(Kcal/d)	2416 ± 677	2308 ± 593	2044 ± 579	< 0.001
Carbohydrate (% of energy)	55.8 ± 7.1	55.7 ± 6.4	56.1 ± 6.9	0.653
protein(% of energy)	13.1 ± 2.3	13.1 ± 2.1	13.5 ± 2.4	0.046
fat(% of energy)	31.0 ± 7.0	31.0 ± 6.3	30.2 ± 7.1	0.264
Saturated fatty acids(% of energy)	4.6 ± 1.4	4.6 ± 1.1	4.5 ± 1.3	0.238
Fiber (g/1000 Kcal)	15.4 ± 7.9	15.9 ± 5.9	17.3 ± 7.1	0.005
fructose(g/1000Kcal)	6.6 ± 2.7	8.0 ± 3.5	9.1 ± 3.6	< 0.001
Vitamin E(mg/1000Kcal)	4.8 ± 1.9	5.0 ± 1.4	5.1 ± 1.5	0.048
**Food groups**				
Whole grains (g/day)	74.5 (27.3–130.9)	56.0 (29.4–112.6)	47.3 (24.4–92.2)	< 0.001
Refined grains (g/day)	381 ± 193	324 ± 147	276 ± 135	< 0.001
High fat dairy products (g/d)	186 ± 150	151 ± 133	125 ± 110	< 0.001
Fruits(g/day)	282 ± 215	351 ± 238	339 ± 225	0.010
Nuts and legume (g/d)	21.37 ± 24.5	23.3 ± 19.5	22.6 ± 24.9	0.560
Red and process meat(g/day)	24.7 ± 19.2	22.8 ± 17.3	20.9 ± 16.9	0.028

Data are shown as Mean ± SD or n (%). Analysis of variance and chi-square test were used for continuous variables for non-continuous variables. Also, to compare the dietary intakes of participants across tertiles of NCV general linear model was used

The association between total NCVs intakes and their subgroups with the odds of NAFLD is presented in Table 2. In the age and sex-adjusted model, the odds of NAFLD was decreased across tertiles of total NCVs (OR:0.36, 95%CI:0.23–0.56), low nitrate vegetable (OR:0.37, 95%CI:0.24–0.58), and high nitrate vegetable intakes (OR: 0.66, 95%CI:0.43–0.99). However, there was no significant association between the medium nitrate intake and the odds of NAFLD. After additional adjustment for BMI, physical activity, smoking, SES, and energy intakes, individuals in the highest vs. those in the lowest tertiles of total and low nitrate vegetables showed a significant lower odds of NAFLD. Furthermore, based on the final model, after additional adjusting for nutritional variables, there is an inverse association between total NCVs (OR:0.20, 95%CI 0.10–0.40; P_trend_ <  0.001) and low nitrate vegetable (OR: 0.22,95%CI 0.11–0.43; P_trend_ <  0.001) and odds of NAFLD.

**Table 2:** Odds ratios (ORs) and 95% confidence intervals (CIs) for NAFLD based on tertiles of nitrat containing vegetables

	Tertiles of NCVs			P-trend
	T1	T2	T3	
**All nitrate containing vegetables**				
Median intake(g/1000Kcal/d)	84.5	148.7	236.6	
NAFLD /control	112 / 148	71 / 152	42 / 150	
Model 1^a^	1.00 (Ref)	0.62 (0.42–0.92)	0.36 (0.23–0.56)	< 0.001
Model 2 ^b^	1.00 (Ref)	0.51 (0.29–0.89)	0.19 (0.10–0.37)	< 0.001
Model 3 ^c^	1.00 (Ref)	0.67 (0.35–1.28)	0.27 (0.13–0.57)	0.001
Model 4^d^	1.00 (Ref)	0.48 (0.27–0.83)	0.20 (0.10–0.40)	< 0.001
**Low-nitrate vegetables**				
Median intake(g/1000Kcal/d)	77.0	135.9	221.2	
NAFLD /control	106 / 150	79 / 150	40 / 150	
Model 1^a^	1.00 (Ref)	0.76(0.52–1.12)	0.37 (0.24–0.58)	< 0.001
Model 2 ^b^	1.00 (Ref)	0.74 (0.42–1.28)	0.20 (0.10–0.40)	< 0.001
Model 3 ^c^	1.00 (Ref)	0.93 (0.48–1.77)	0.29 (0.14–0.61)	0.001
Model 4^d^	1.00 (Ref)	0.68 (0.39–1.19)	0.22 (0.11–0.43)	< 0.001
**Medium-nitrate vegetables**				
Median intake(g/1000Kcal/d)	0.04	0.62	2.22	
NAFLD /control	80/149	78/151	67/150	
Model 1^a^	1.00 (Ref)	0.94(0.64–1.40)	0.79 (0.53–1.19)	0.252
Model 2 ^b^	1.00 (Ref)	1.50 (0.85–2.64)	0.90 (0.50–1.63)	0.430
Model 3 ^c^	1.00 (Ref)	1.94 (0.99–3.77)	1.10 (0.56–2.17)	0.812
Model 4^d^	1.00 (Ref)	1.47 (0.84–2.57)	0.96 (0.53–1.71)	0.574
**High-nitrate vegetables**				
Median intake(g/1000Kcal/d)	3.45	7.67	15.14	
NAFLD /control	87/145	78/154	60/151	
Model 1^a^	1.00 (Ref)	0.85(0.58–1.26)	0.66 (0.43–0.99)	0.048
Model 2 ^b^	1.00 (Ref)	0.65 (0.37–1.14)	0.60 (0.33–1.09)	0.121
Model 3 ^c^	1.00 (Ref)	0.64 (0.33–1.23)	0.81 (0.40–1.64)	0.707
Model 4^d^	1.00 (Ref)	0.63 (0.36–1.10)	0.62 (0.34–1.12)	0.156

^a^Model 1: adjusted for age and sex

^b^Model 2: Additionally adjusted for BMI, physical activity, smoking, SES, and dietary intake of energy

^c^ Model 3: adjusted for model 2 and refined grains, high-fat dairy, red and processed meats, fruits except for melon and watermelon

^d^ Model 4: adjusted for model 2 and dietary intake of saturated fatty acids, vitamin E, fiber, and fructose

Table 3 shows the odds of NAFLD per one SD increase in the total dietary nitrate intake from various food groups. The final model indicated that each one SD increments in the total nitrate were not related to the odds of NAFLD. Each one SD increments in nitrate content of vegetables (OR:0.73,95%CI:0.55–0.97;*P* = 0.034), nitrate of fruits (OR:0.59, 95%CI:0.36–0.97;*P* = 0.040) was associated with decreased the odds of NAFLD. However, there was a positive association between each one SD increments in nitrate content of dairy products and meats and processed meats with odds of NAFLD (OR:1.34,95%CI:1.03–1.74; *P* = 0.025).

**Table 3:** The odds ratio and 95% confidence interval for the risk of NAFLD per on SD increase in dietary total nitrate and nitrate intake from food groups among study participants

	OR (95% CI)	*P* value
**Total nitrate**		
SD(mg/d)	175.0	
Model 1^a^	1.10 (0.93–1.28)	0.240
Model 2^b^	0.76 (0.57–1.00)	0.056
Model 3^c^	0.79 (0.57–1.10)	0.178
**Nitrate content of vegetables**		
SD(mg/d)	124.5	
Model 1^a^	0.93 (0.79–1.11)	0.464
Model 2^b^	0.70 (0.53–0.92)	0.012
Model 3^c^	0.73 (0.55–0.97)	0.034
**Nitrate content of fruits**		
SD(mg/d)	44.9	
Model 1^a^	0.96 (0.76–1.22)	0.773
Model 2^b^	0.59 (0.41–0.86)	0.006
Model 3^c^	0.59 (0.36–0.97)	0.040
Nitrate content of grains, legumes, and nuts		
SD(mg/d)	69.2	
Model 1^a^	1.28 (1.09–1.50)	0.002
Model 2^b^	1.29 (0.99–1.68)	0.059
Model 3^c^	1.37 (0.91–2.06)	0.125
Nitrate content of dairy products and meats and processed meats(mg/d)		
SD	9.5	
Model 1^a^	1.45 (1.23–1.72)	< 0.001
Model 2^b^	1.27 (1.00–1.59)	0.042
Model 3^c^	1.34 (1.03–1.74)	0.025

^a^Adjusted for age and sex

^b^Additionally adjusted for BMI, physical activity, smoking, SES, and dietary intake of energy

^c^adjusted for model 2 and dietary intake of saturated fatty acids, vitamin E, fiber, and fructose

## Discussion

In this case-control study, we showed that a high intake of total nitrate-containing vegetables and low-nitrate-containing vegetables was related to decreased odds of NAFLD, independent of potential confounding variables. In contrast, there was no significant association between intakes of medium- and high nitrate-containing vegetables and the odds of NAFLD. Also, our finding reported that one SD increment in nitrate content of vegetables and fruits was linked inversely with odds of NAFLD; however, a positive association was observed between each one SD increments in nitrate content of dairy products and meats and processed meats with risk of NAFLD. Furthermore, each one SD increments in the intakes of all dietary nitrate had no association with odds of NAFLD.

To date, this study was the first study that examines the relationship of NCVs with the odds of NAFLD; however, several studies have previously assessed the association of dietary nitrate intake or NCVs with the risk of other chronic diseases such as CKD [16], atherosclerosis and ischemic cerebrovascular disease [17], blood pressure [8], and CVD [18], which all of them have the characteristics of metabolic disorders similar to NAFLD. A prospective study conducted on Tehranian adults has reported that high intake of high-NCVs was associated with increased the risk of CKD at baseline, but, after 3 years of follow-up, no significant association was observed between intakes of total NCVs and its categories (high-, medium-, and low NCVs) with the risk of CKD [16]. However, in a population-based cohort study, Golzarand et al. have shown that dietary nitrate from vegetable sources can significantly decrease the risk of hypertension (HTN) and consequent CVD complications, independent of dietary fiber and potassium [8]. Also, Bondonno et al. have revealed that older women who had higher intakes of vegetable nitrate had a significantly lower risk of carotid atherosclerosis and ischemic cerebrovascular disease event [17]. Furthermore, the Australian Longitudinal Study has provided evidence on the protective role of vegetable nitrate intakes on developing CVD-related disorders, including HTN, thrombosis, heart disease, and stroke among middle-aged Australian women over a 15-year follow-up period [18]. Although studies conducted on total dietary nitrate intake or NCVs have examined their role in the risk of developing various chronic diseases, which may also be methodologically different, the findings of most of these studies provided evidence that the intakes of nitrate from vegetable sources can have a beneficial impact on the prevention of developing chronic diseases such as NAFLD, HTN, CVD complications.

Convincing evidence has suggested that individuals with higher consumption of vegetables had a lower risk of NAFLD [25, 26] . This process may be associated with higher intakes of fiber, antioxidant vitamins, and bioactive compounds, as well as the low energy content of vegetables. According to interesting findings of the current study, we also suggested that the high consumption of total NCVs has been remarkably associated with a reduction in the odds of NAFLD, which can be due to increased intake of dietary nitrate; because, in the current study, the inverse association between NCVs and risk of NAFLD was reported and confirmed independently of other dietary factors such as dietary intakes of energy, refined grains, high-fat dairy, red and processed meats, fruits, saturated fatty acids, vitamin E, fiber, and fructose, each of which could affect the risk of NAFLD. So, these results of our study show that nitrate intake from vegetables not only does not increase the risk of NAFLD but may also be a protective factor in the form of vegetables against risk of chronic diseases such as NAFLD. It should be noted that, according to the classification of vegetables into three groups based on nitrate content, although the odds of NAFLD is reduced with increasing intake of vegetables in all three groups, the results are significant only in the group of low nitrate vegetables. The main reason that the results of the group of low-nitrate vegetables have been remarkable compared to the other two groups (medium-nitrate vegetables and high-nitrate vegetables groups) is that, according to the definition of NCVs, most of the total vegetables intakes in our study population was low nitrates vegetables (more than 90% of all vegetables intakes based on the results of Table 2), and so the individual’s dietary intakes of medium-nitrate vegetables and high-nitrate vegetables was very low. Also, there is no high dispersion in the dietary intake of vegetables in participants across tertiles of the medium-nitrate vegetables and high-nitrate vegetables groups, as a result, the power of medium-nitrate vegetables and high-nitrate vegetables has not been significant in predicting the odds of NAFLD compared to low-nitrate vegetables group.

Also, we showed that differences in dietary sources of dietary nitrate could also play a significant role in the risk of liver disease; our results reported that higher intake of nitrate from plant-based food sources, including vegetables and fruits, have a protective role against onset or progression of NAFLD, whereas, higher intake of nitrate from animal-based food sources including dairy products and meats and processed meats have worsened the development of NAFLD. Therefore, our finding in Table 3 suggested that the nature of food groups and their related characteristics can cause nitrate intake to play a different role in predicting the risk of NAFLD. In other words, it seems that differences in food sources of nitrate have an influential role in selecting the target pathway for nitrate to enter the metabolic cycle.; this in turn subsequently causes dietary nitrate intake to play a dual role in (increasing or reducing) the odds of NAFLD. Based on recent documents, the potential physiological and pathological role of nitrate intakes from various food sources in the body are not fully understood, and also there are controversial views on the possible adverse impact vs. health benefits of nitrate intake [11, 12, 27]; although, further studies are needed to understand how dietary nitrate intake is metabolized within the food matrix, it seems that on a metabolic level this paradoxical role of nitrate intakes is possibly not surprising, and it is expectable that all food sources of nitrate are not necessarily identical with regards to their potential health impacts [28]. Previous studies have reported that the inducing production of nitric oxide (NO), a gas produced by nitric oxide synthase (NOS) enzyme, slows NAFLD development [29], and also the reduction or deficiency of eNOS accelerate early-stage NAFLD progression via alteration of the fat distribution [30]. The bioactivity of nitrate, nitrite, and NO metabolism is a complicated process that can be affected by the nature of the consumed foods and their nutrients. The higher intakes of pro-inflammatory nutrients, including saturated fat, sodium, and amine compounds from animal food sources such as dairy foods, meat, and processed meat may interfere with eNOS and decreased the NO production from nitrate [18], however, the higher intakes of anti-inflammatory compounds including polyphenols, fiber, vitamin C, and other antioxidants from vegetables and fruits can have enhancing role on NO production from nitrate [31–33]. Also, it has been reported that higher intakes of nitrate/nitrite from vegetables may contribute to improving cardiometabolic conditions via beneficial impact on insulin signaling pathway, insulin resistance, and inflammation and oxidative stress [11].

The current study had several strengths. This study is the first study that assesses the association of all dietary nitrate intakes and all NCVs with odds of NAFLD. We also analyzed the role of three categories of NCVs consumption based on nitrate content (low-, medium-, and high NCVs) with NAFLD. In addition, the relationship of nitrate intakes from various other food groups (fruits, grains, legumes, nuts, dairy products, red meats, and processed meats) with odds of NAFLD was examined to provide more explanation on the role of nitrate intake in the development of NAFLD based on different food sources. We have used a validated and reproducible FFQ to collect participants’ dietary data, which was completed by trained dietitians in a face-to-face interview; this questionnaire was not a self-reported questionnaire that led to minimizing measurement bias. Furthermore, we used a validated questionnaire to assess the physical activity levels of individuals.

Despite the above-listed strengths, some limitations deserve to mention. First, this study cannot discover the causality between exposures and outcomes because of the case-control design. Second, we used ultrasonography test to diagnose NAFLD in participants, but a biopsy of the liver and magnetic resonance imaging (MRI) technique is the gold standard and more accurate tests for diagnosis of NAFLD; it is worth mentioning that because of the limitations and complications of biopsy and high cost and low availability of MRI, using noninvasive methods such as ultrasonography is applicable and reliable to diagnosis NAFLD in clinical practice [34]. Furthermore, in this case-control study, the matching process of the cases and controls was not performed based on main variables including age, sex, and BMI, because it could make it more difficult to select eligible individuals and lengthen the sampling time. Also, it could increase the possibility of over-matching; however, it should be noted based on baseline results, age and sex distribution did not differ in participants based on cases and controls groups and also the possible confounding role of these variables such as BMI were controlled in the multivariable-adjusted analysis. Moreover, some inherent limitations of case-control studies, including selection bias and information bias in determining exposure or outcome, should be considered in interpreting the results. Finally, despite the adjustment of various potential confounders, our study design cannot eliminate all possible confounders, and the effects of some residual confounders may have occurred.

## Conclusions

In conclusion, our results presented evidence for an inverse relationship of all-and low NCVs intakes with odds of NAFLD. However, no significant association was observed between medium- and high NCVs and NAFLD. In addition, we reported that one SD increment in nitrate content of vegetables and fruits could be had a beneficial impact in reducing the odds of NAFLD, but there is a direct link between higher intake of Nitrate content of dairy products and meats and processed meats with odds of NAFLD. Our finding suggested that differences in sources of dietary nitrate are led to this compound play a different role in the beginning or progression of NAFLD. Further research is needed to clarify the possible role of nitrate intakes from various sources of foods on liver function.

## Data Availability

The data underlying this article will be shared at reasonable request to the corresponding author.
